# From reductive to generative crisis: businesspeople using polysemous justifications to make sense of COVID-19

**DOI:** 10.1057/s41290-021-00147-w

**Published:** 2022-01-19

**Authors:** Ioana Sendroiu

**Affiliations:** 1 Weatherhead Center for International Affairs, Harvard University, 1737 Cambridge Street, Cambridge, MA 02138 USA; 2 Max Planck Institute for Research on Collective Goods, Kurt-Schumacher-Straße 10, 53113 Bonn, Germany

**Keywords:** COVID-19, Crisis, Justifications, Polysemy, Meaning-making

## Abstract

Both lay understandings of crisis moments and influential psychological models of cognition in times of uncertainty emphasize how crises limit thinking. Conversely, scholars as diverse as Foucault, Swidler, Bourdieu, and Butler have elaborated generative conceptions of crisis, which specify crises as moments of change, transformation, and heightened cognition. The research presented here takes up the question of how crises become thinkable, as actors gradually make sense of a newly uncertain context. Against a backdrop of polarization on the topic, in-depth interviews with 60 businesspeople navigating the coronavirus pandemic show that they see public health and economic well-being as interrelated. This has important effects on how businesses interpret and implement government directives and public health guidelines, from choosing to close before being mandated to do so, to staying closed even when allowed to reopen. Taken together, these findings substantiate generative models of crisis while drawing attention to the polysemous justifications elaborated by actors as they navigate shifting cultural and social scaffoldings.

## Introduction

During the 2020 US presidential election, exit polls, according to the *New York Times*, “showed the vote came down to the pandemic versus the economy” (Medina and Russonello 2020). More than any other issue, preferred modes for handling the pandemic determined voting choices. Those concerned about rising infections, who viewed managing COVID through a public health lens, voted for Joe Biden. Those concerned about the financial ramifications of widespread economic shutdowns, who were in favor of reopening the economy, voted for Donald Trump. These two perspectives—privileging public health versus focusing on economic well-being ostensibly—did not coincide. Given the “dual national crisis” along both health and economic fronts, exit polls showed that “voters were deeply divided on what mattered more” (Medina and Russonello 2020).

Such polarization is of course now common in the United States (Iyengar et al. 2019). But this unambiguous choice of one perspective over another also fits with psychological models of cognition in times of crisis. Indeed, a large body of research from psychology suggests that situations of uncertainty produce shifts in how actors make sense of the world around them, simplifying cognition and limiting willingness to consider multiple interpretations of ambiguous information (for a review, see Kahneman and Klein 2009). In particular, moments of crisis, which are marked by widespread uncertainty, are said to simplify cognition and decision-making, with this view having been explicitly linked to how individuals are making—or failing to make—sense of COVID-19 (e.g., Chater 2020).

In the midst of this economic and public health crisis, the research presented here investigates how businesspeople are themselves making sense of the tradeoffs between financial well-being and public health. This is all the more important because political decisions to (re)open economic activity, particularly when done against the advice of public health guidelines, are based on an economy-first logic. Put differently, they are based on the view that individuals, and especially people in business, care most about their financial well-being. And indeed, facing existential threat, these businesses would be well justified to focus on their economic survival (on survival as opposed to profit maximization, see Fligstein 2001).

Yet, considerable work in economic and cultural sociology has shown that markets are profoundly moral (Fourcade and Healy 2007), and that economic actors define themselves in ways that expand well beyond profit maximization (Fligstein 2001; Spillman 2012). But none of this research has explicitly considered moments of crisis, when actors are generally more likely to seek to reduce ambiguity (Kahneman and Klein 2009), and when they are facing existential threats. Indeed, the economic effects of COVID-19 are devastating, with unemployment rates at record highs and estimates that many businesses, especially small ones, will not be able to survive the pandemic (Aaronson and Alba 2020; Casselman et al. 2020).

In what follows, these perspectives are brought to bear on the question of how economic actors—in particular, people in business—are making sense of, and responding to the COVID-19 crisis. In-depth interviews conducted in April and May 2020 focused on how 60 businesspeople managed economic imperatives while dealing with the public health effects of the crisis. Indeed, as part of the in-depth interviews, respondents were asked to elaborate and think through the matter of economic well-being versus public health. The interviews show that businesspeople negate a stark choice between the two, with these sophisticated views guiding reactions to the crisis.

This is partly because businesspeople themselves identify goals and values that take primacy over economic survival, particularly public health. But at the same time, of course, businesses are concerned about their economic well-being. Crucially, the two are not seen as mutually exclusive. People in business see both the economic effects of a prolonged public health crisis, and the ways in which an economy doing poorly can affect public health. In turn, actions taken to navigate the crisis are explained in terms of both economic survival and the protection of the communities that businesses serve. In particular, these views affect how businesses interpret and implement government or public health guidelines, and more generally how they take unprecedented action in response to the crisis, spanning from deciding to close before being mandated to do so, to refusing to reopen even when allowed.

This suggests a polysemous model of crisis that foregrounds a multiplicity of frames being elaborated by actors to justify the actions they take during crisis. Such a model draws on generative conceptions of crises as changing cultural and social scaffoldings that throw dispositions into disarray and heighten cultural articulation (Bourdieu 1990, 2014; Butler 1993, 1999; Foucault 1997, 2000; Luft 2020; Swidler 1986). But the findings presented here go further in specifying that these efforts to make sense of an unsettled moment are marked by a multiplicity of competing and compelling frames for understanding the shifting context (see also Boltanski and Thévenot 1983; 2006 [1991]), a process that can help render a crisis “thinkable” (Alexander and Smith 2020: 264).

Therefore, rather than simplification in response to uncertainty and threat, crises can also allow for polysemous interpretations as actors seek to navigate changing cultural and social scaffoldings. For businesses navigating COVID-19, prosocial behavior can of course be justified as a matter of public protection, but also in terms of profit-seeking. In this way, moral action can prevail even during instances of existential threat, when moral choices are both unclear and potentially carry great risk.

## Moral markets during COVID-19?

Self-interested, profit-maximizing, rational action models of economic behavior have been increasingly challenged in recent years, for assuming that individuals’ goals are well defined, or that “rational” behavior will involve finding the most effective means to realize these goals (Granovetter 2017; Hirschman 2013 [1977], 1982). In fact, the concept of a moral economy emerged in the 1970s to foreground the set of norms or values that govern economic behavior, and delimit what counts as morally appropriate economic behavior (Granovetter 2017; Thompson 1971). Increasingly, a growing body of work in economic sociology has even rejected the existence of a distinction between moral norms and the market, arguing instead that markets are both moralized and moralizing entities (Abend 2014; Fourcade 2011; Fourcade and Healy 2007; Zelizer 2010).

These academic debates have taken on new significance during the COVID-19 pandemic. In a moment when everyone is called on to “think more socially” than ever (Mull 2020), and “[o]ur responses must be altruistic to save as many as possible” (Luft 2020: 2), the likelihood, ability, and willingness of economic actors to act “morally” and prosocially becomes a crucial shaper of how they navigate the pandemic (see also Tooze 2020). Simply put: during the pandemic, self-interested, profit-maximizing action aiming for economic survival is often conceived to be starkly at odds with the need to close down market economies in order to mitigate the spread of coronavirus. So can markets remain moral and moralizing in contexts of economic devastation and existential threat?

There is reason to believe that people in business will act prosocially, or at least not solely privilege profit maximization. Past research has shown that people in business do not think of, nor conduct their work as profit-maximizing, self-interested action (Spillman 1999; 2012). Instead, businesspeople “often think of what they are doing in terms of technical expertise, professionalism, stewardship of the public good and occupational community, and these vocabularies of motive are a constitutive part of many business identities” (Spillman 2012: 2). In particular, these sorts of prosocial motivations can underpin strategic interest by making economic action meaningful for those involved, rather than negating competitive profit-seeking entirely.^1^ Past research into business behavior during natural disasters underscores these findings (Chen et al. 2013; Doern et al. 2019; Dutta 2017); for instance, “big-box” retailers such as Walmart helped mobilize essential resources in the aftermath of Hurricane Katrina (Rosegrant 2007).

But COVID-19 may nonetheless have changed the moral calculus of markets, or indeed may have entirely thrown typical business moral norms into disarray, with the unsettled context making it so that usual norms functioned poorly to guide behavior. The pandemic was unprecedented in a way that natural disasters are not, mandating a desperate scramble for survival (a goal that indeed defines the sociology of markets, see Fligstein 2001). Thus, given the widespread economic devastation, with many businesses—especially small ones—facing bankruptcy, and the lack of clarity on the best choice of prosocial action, businesses could try to survive the crisis by limiting prosocial behavior (Aaronson and Alba 2020; Casselman et al. 2020). Put differently, we know that markets are moral and moralizing during settled times, but is this still the case during crisis, when economic actors face acute threats to their continued existence and have little sense of how best to address these threats?

## Models of reductive crisis cognition

Considerable work in psychology links uncertainty to the increased use of cognitive shortcuts, known as heuristics (e.g., Kahneman et al. 1982). This is particularly the case during crises, moments marked by widespread and deeply felt uncertainty. The influential heuristics and biases approach most associated with Tversky and Kahneman (e.g., Tversky and Kahneman 1973; 1974) details the many ways uncertainty limits cognition (see also Fischhoff and Broomell 2020; Gigerenzer and Gaissmaier 2011; Gilovich et al. 2002). Even expert knowledge is shown to be faulty; indeed, detailing the limits of expert intuition has been part of the heuristics and biases approach from the start (Tversky and Kahneman 1971).

Outside of the laboratory experiments preferred by researchers in the heuristics and biases tradition, we can again see the limits of cognition during conditions of uncertainty (Kahneman and Klein 2009). For instance, the naturalistic decision-making approach has long focused on decision-making during conditions of uncertainty, interested in how experts can often be successful in managing uncertainty. An early study looked at how fireground commanders make decisions, with Klein et al. (1986) showing that commanders only generated one option for behavior, drawing on a vast repertoire of patterns they had accumulated during their years of service. The commanders would then mentally evaluate the one option, looking to whether modifications would be necessary, but generally just stuck with the one option. In contrast with the heuristics and biases approach, this research showed that fireground commanders’ focused cognition strategy was effective because it took advantage of commanders’ ability to assess a new problem with reference to past situations they had already faced. And indeed, subsequent work in the naturalistic decision-making tradition has followed along these lines, detailing—and seeking to demystify—the cues experts use to make decisions, even as these cues are not often obvious (e.g., Crandall et al. 2006; Schraagen et al. 2000).

But even the naturalistic decision-making approach concedes that expert intuitions will be ineffective in environments that are “insufficiently predictable” or in the “absence of opportunities” to learn the rules of an environment (Kahneman and Klein 2009: 521). Expert intuition can be remarkably effective at managing uncertainty, but only insofar as experts have already experienced situations of uncertainty that have similar contours to the one they are currently facing. Indeed, the naturalistic decision-making approach shows that even effective uncertainty management involves substantial cognitive simplification—after all, fireground commanders typically considered only one option for behavior (Klein et al. 1986).^2^ Thus, given that crises are moments of change when old rules and patterns no longer apply, there is certainly an expectation of limits and shortcuts imposed on cognition and decision-making during crisis.

This sort of cognitive narrowing, it should be noted, is not time-limited to the initial moment when a crisis is recognized or performed into being. For instance, psychologists have detailed how uncertainty continues to limit environmental protection despite well-developed knowledge on how climate change can be addressed (Gifford 2011; Gifford and Chen 2017; Lacroix and Gifford 2018; see also Stern 1992). Of course political ideology matters too, as do other biases, but uncertainty is an integral component of actors' reducted cognition in response to a long-standing crisis such as climate change (Budescu et al. 2009; de Kwaadsteniet 2007; Hine & Gifford 1996).

But at the time of this study (spring 2020), COVID-19 was not even a long-standing crisis, the virus having emerged just months prior. While the initial shock of the pandemic may have worn off, the uncertainty was still deeply felt regarding how to interpret the pandemic, or how best to react. Put differently, businesspeople were still very much in crisis. Indeed, the view of crisis as simplifying cognition was explicitly linked to COVID-19 (Barnett et al. 2020; Berger et al. 2021; Chater 2020; Hahn et al. 2020; Lordan et al. 2020). Writing in *Nature*, psychologist Nich Chater (2020) argued that because “we know so little about the virus…our brains struggle to organize this confusing mass of partial and jumbled information into a coherent interpretation. And we make decisions as if that interpretation is true, without entertaining alternatives.” Collectively, this could have devastating consequences for managing the crisis (Chater 2020), particularly for policymakers struggling with deeply felt uncertainty even a year into the pandemic (Berger et al. 2021; see also Barnett et al. 2020). Meanwhile, for individual economic actors, an inability to shift among interpretations of the crisis could limit capacities to respond effectively and creatively to the situation (see also Boltanski and Thévenot 2006 [1991]; Stark 2009).

## Generative views of crisis

Work in sociology has also dealt with how actors orient action during crisis, typically defined as unsettled times marked by widespread uncertainty when existing cultural scaffoldings no longer work effectively to guide behavior (Bourdieu 1990; Lizardo and Strand 2010; Swidler 1986). But sociological theorizing on crisis sees such moments as generative rather than reductive. Indeed, despite their differences, the picture of crisis that emerges from both Swidler’s (1986) writing on unsettled times and Bourdieu’s (1990, 2000, 2014) on moments of social change is one of changing social and cultural structures setting of a period of discomfort as individuals’ toolkits or habitus no longer work well for a new context. This eventually engendering some form of behavioral or cultural change by the individuals themselves (see also Lizardo and Strand 2010; Luft 2020). In this sense, crisis moments have been conceptualized as important drivers of changing behavior, whether in cultural approaches (Bourdieu 1990; Swidler 1986; see also Lizardo and Strand 2010), or in political sociology work focusing on structural change, particularly through the prism of events (Abbott 2001; Sewell 1992, 1996, 2005; Wagner-Pacifici 2017).

This productive view of crisis can also be found in the work of post-structuralist scholars such as Butler (e.g., 1993, 1999) or Foucault (e.g., 1997, 2000), who situate crisis as a starting point of critique, and therefore of transformation (see also Roitman 2005, 2014). As Butler (2002: 215) explains upon grappling with Foucault’s attempts to explain the basis of critique, “[o]ne asks about the limits of knowing because one has already run up against a crisis within the epistemological field within which one lives.” The basis of critique, then, is a crisis rupture (see also Koselleck  2000). And indeed, conversely, the role of critique is to produce crisis (Foucault 1997).

Throughout these generative models, the precursor to behavioral change in response to crisis is a sort of heightened visibility or explicitness of culture. Swidler (1986: 284) thus defines unsettled cultural periods as moments when “cultural meanings are more highly articulated and explicit, because they model patterns of action that do not ‘come naturally.’” Similarly, in his writing on state formation and change, Bourdieu (2014: 30) argues that in such moments, actors must come to the fore who reconcile the group to which they belong with a new “official truth” that comes out of, and is elaborated through, the crisis moment itself. Meanwhile, in post-structuralist accounts of crisis, “while truth cannot be secured, it is nonetheless performed in moments of crisis, when the grounds for truth claims are supposedly made bare and the limits of intelligibility are potentially subverted and transgressed” (Roitman 2014: 34).

But beyond this general consensus around the greater explicitness of culture during crisis moments, we know less about how actors attempt to make sense of crisis and what underpins these heightened levels of articulation, or the post-structuralists’ renewed search for truth. It is here that this paper intervenes, building on the generative models of crisis with a focus to how actors juggle multiple justificatory frames when navigating crisis moments. If indeed crisis can be generative, then we should be able to detect this in how actors juggle justificatory frames in order to make sense of their actions. Thus, precisely through this process of “highspeed bricolage” across polysemous justifications, an unprecedented, difficult-to-understand event such as COVID-19 can—rather than limiting cognition—become “thinkable” (Alexander and Smith 2020: 264).

## Polysemous crisis justifications

In elaborating this polysemous conception of crisis, I draw on Boltanski and Thévenot's (2006 [1991]) theory of justifications (see also Lamont 2012).^3^ Simply put, they theorize that actors consistently shift among modes for justifying and evaluating their interests and goals (Boltanski and Thévenot 1983, 2006 [1991]). This helps solve the question above, regarding the cultural mechanisms underpinning the generative view of crisis.

Indeed, Boltanski and Thévenot elaborated their model in response to Bourdieu’s more unitary view of habitus, a view that is also implicitly part of Bourdieu’s model of crises. For instance, in *Homo Academicus*, Bourdieu (1990) details the struggles among different actors using a variety of frames or perspectives in order to compete for prominence during the 1968 protests in France. But these frames emerge out of these actors’ positions within pre-crisis fields: tenured professors take a perspective on the protests that differs from nontenured academics, representatives of less prestigious disciplines (such as sociology!) are more amenable to the protests, and so on (Bourdieu 1990; see also Strand and Lizardo 2017). For Bourdieu, then, these competing frames are rooted in actors’ structural positions, rather than any one actor having the ability to wield competing justifications.

Boltanski and Thévenot (2006 [1991]) meanwhile argue that actors justify their positions by appealing to different (and diverse) logics; for instance focusing on profit maximization when making use of a market logic, or emphasizing civic solidarity when engaging with a civic logic. In turn, subsequent empirical research has shown that actors will draw from a range of such logics in justifying their claims to money and recognition (Levi and Sendroiu 2019). A multiplicity of grammars of worth can even explain diversity in definitions of personal worth, which Lamont (1994; 2009) operationalizes in terms of money, morals, and manners.

And this range of justifications could be particularly important in situations marked by change. Research on the high-tech sector shows that renegotiating and shifting justifications is essential for innovation (Stark 2011). Similarly, Boltanski and Thévenot (2006 [1991]) theorize that as actors navigate shifting environments, they must renegotiate and reconstruct the logics they employ. This is the case to such an extent that Boltanski and Thévenot (2006 [1991]: 350) explicitly define crisis in terms of the wielding of divergent frames as part of a common project to define a new reality: “[I]n the concept of crisis, we have chosen to retain not a moment of chaos created by actors following their own separate paths with no attempt to coordinate their actions, but moments when partners agree on the need to define the reality that they have to take into account.” For them, attempts at justifying a new reality *are* the crisis.^4^ Indeed, Stark (2011) shows that such justificatory work can effectively turn uncertainty into risk, which suggests attempts to manage and even move past the crisis moment.^5^

Bringing together these models of crisis with work from economic and cultural sociology that views markets as moral, the empirical goal here is to explore how people in business are understanding and responding to the COVID-19 crisis. During a crisis when many businesses are facing existential threat, is there a shift to focusing just on economic survival, or do actors wield polysemous justifications to make sense of what is happening?

In what follows, interviews with 60 businesspeople in Canada and the US are used to elaborate the justifications people in business use to make sense of the crisis, and to guide their reactions to the pandemic. The interviews show that even during COVID-19, people in business did not view economy and public health as mutually exclusive. As a result, their actions took into account both their businesses' survival and other moral considerations such as the protection of their communities, customers, and staff. This substantiates the view of crises as polysemous. For economic actors, the existential threat posed by a crisis such as COVID-19 does not mean focusing in on one matter, such as profit, but rather that action taken in reaction to crisis will be underpinned by a variety of justifications.

## Methods

Interviews took place between April 12 and May 11, 2020, involving 35 businesspeople in the United States and 25 in Canada (a more expansive consideration of the split Canada/US sample can be found in the Appendix). The interviews were done on the phone or online, according to the interviewees’ preferences. On request from the respondents, 5 of the interviews took place over email. In total, 58 interviews were conducted, which represented 57 businesses, and involved 60 respondents: two individuals from the same business were interviewed, and two further interviews involved the simultaneous interviewing of business partners.

On average, interviews lasted 35 minutes, and ranged in length from 11 minutes to an hour (the 11-minute interview was cut short by the respondent due to a personal matter and subsequent questions were answered over email). The interviews were semi-structured, focusing on how respondents’ businesses reacted to COVID-19, as well as their views and plans for the future. Case-specific follow-up questions were also asked to get a fuller view of businesses’ experiences.

Interviews inadvertently captured the range of life under COVID-19 lockdown. Some respondents were at home and able to speak though video call. Others were working at the same time as doing the interview, doing everything from roasting coffee to smoking fish. Interviews were also paused for a range of reasons, from curbside deliveries to rowdy kids.

Roughly 1272 businesses were invited to participate in the interviews (this is a rough estimate because it is unclear whether all participation requests were received). Businesses were contacted if they were featured online in relation to COVID-19, whether in newspaper articles on COVID-19, lists of suppliers of COVID-19-related goods, lists of businesses struggling with the effects of the crisis and asking for donations, or lists of businesses aiding others during the crisis.

The inclusion criteria, meanwhile, extended to anyone involved in decision-making on behalf of a business in Canada or the US during the COVID-19 crisis. Nonetheless, the interviews are evidently not a representative sample of businesses in either country. The goal of this study was rather to gain an in-depth understanding of how people in business were thinking through the challenges posed by the COVID-19 crisis. Beyond the broader, more representative data created through large-N surveys, in-depth interviews afford an opportunity to probe how respondents are not just acting in response to COVID-19, but also justifying their actions (Lamont and Swidler 2014).

The sampling was therefore purposive, with the goal of seeking out a breadth of experiences and contexts. In terms of contexts, interviews included places where the economy was reopening (such as Georgia/US or Nova Scotia/Canada) or not (Washington/US); places with many infections (New York/US) or few (British Columbia/Canada); and places with growing infections (Quebec/Canada) or decreasing rates of infections (Alberta/Canada). A breadth of business types was also sought out, from businesses that were forced to close, such as yoga studios, to those deemed essential, such as toilet paper suppliers, and from businesses hard hit by the economic shutdown, such as restaurants, to those experiencing a boom in business, such as those manufacturing feminine hygiene products. The sample thus included businesses both struggling and thriving as a result of the pandemic.

While the sampling criteria were not limited to particular business sizes, no large businesses responded to interview requests. The sample is therefore made up predominantly of small businesses. But given that the pandemic more strongly threatened small businesses (Aaronson and Alba 2020; Casselman et al. 2020), this is precisely the population among which we would aim to study how economic actors manage extreme uncertainty and existential threat. However, future analyses should certainly consider a wider range of business sizes.

In order to preserve confidentiality, interviews are identified according to the state or province where the business is located, as well as the business type. Table 1 includes a breakdown by business types. 4 interviews were done with yoga studios and gyms, which were coded as fitness services. 26 interviews focused on food service businesses, ranging from James Beard Award-winning restaurants to board game cafes. Businesses such as campgrounds or golf courses were coded as leisure services (N=4). 2 more businesses were manufacturers, while 5 other interviewees also sold what they manufactured directly to consumers, and so were coded as “manufacturing and retail.” Shops and stores were coded as “retail” (N=7). Finally, a further 12 businesses were coded under a broader category of “services,” and ranged from massage therapy and hair salons to a doula.

**Table 1:** Distribution of business types

*Business type*	*N interviews*
Fitness services	4
Food services	26
Leisure services	4
Manufacturing and retail	7
Retail	7
Services	12

## Results

### Decisions to close: “I'm so slow and I'm sitting here and I'm stressing out.”

The interviews cover the gamut of experiences, from closing permanently and declaring bankruptcy due to the crisis, to thriving by nature of the products sold or being declared essential. The interviews happened as widespread shutdowns were mandated across most of North America, though almost all businesses interviewed were continuing economic activity, generally in reduced or modified capacity. A third of those interviewed chose to close before being mandated to do so, a decision that was notably explained both in terms of moral commitments to helping protect public health, as well as an attempt to manage decreasing profits.

The reasons given for closing early always referenced public health. Respondents insisted that clients are “friends” (US/Connecticut, retail) or “neighbors” (US/Washington, food services) and so businesspeople have a responsibility to protect them. Businesspeople were also concerned about the health of their employees, or their own health. A number of businesses even reported that employees demanded that they close, with one businessperson telling the story of a staff member who simply stopped showing up at work (US/Connecticut, retail).

Frequently, decisions to close early were couched in moral terms. The following statement from a California retail businesswoman captures this well:

I think I had started the cleanliness, you know, things like that, the weekend or the half week before we were forced to close. Then I stayed open on that Monday. I think that Monday night I was having a lot of anxiety and stress. I couldn't sleep. I would keep teeth grinding. It was like crazy. I saw my other local businesses that shut their doors, and I was like, “*This is selfish*, I need to shut my doors.” I think I made a last-minute announcement on that Monday, “I'm closing it.”

In this case, staying open given the growing crisis was seen as “selfish,” particularly when other businesses had already begun closing. For this respondent, closing was therefore the moral choice, with staying open during the public health crisis causing considerable anxiety.

The interview was then briefly interrupted so that the respondent could complete a curbside pickup. When she came back, she was asked to expand on the source of her anxiety, and she said that she was worried about herself and her staff. In particular, the respondent felt that customers were not taking social distancing seriously: “customers were coming in and super casual about it. Really it's not a big deal, all that stuff....I was upset because someone did cough....It was just one of those things [where] you're like, well, I just don't want to risk anything from anyone.”

This concern about reckless customers matches many other interviews, where businesspeople reported being worried about their staff’s health because customers were acting in threatening ways. For instance, a Georgia coffee shop that stayed open for pickup had to call the police when a longtime customer came in to order food, mentioned that he had been sick, and refused to leave the premises. Or, before restrictions on dine-in service were implemented, one Manitoba coffee shop had customers with luggage come in to the premises. After being served, they mentioned that they had just flown in from the States and wanted to “pass the time” at the shop before heading home, in defiance of the mandatory 14-day quarantine that had already been implemented in Canada at that point.

Indeed, not encouraging customers to spread the virus was also couched in moral, public health terms. For instance, the owner of a brunch spot in Ontario, Canada recounted that she closed as she became increasingly frustrated with her customers for continuing to socialize:

[W]e were all just going about our regular daily business and then it started to feel like it was getting a lot more serious. I made the decision on March 15th, which was a Sunday, to pretty much say, “You know what? I don't feel safe anymore. I don't feel safe for my employees. I don't feel safe for myself,” and I didn't feel like it was a morally right thing to do to keep my doors open and encourage my customers to come out and socialize and brunch.

Indeed, right before closing, her business had “a really busy weekend” and she recounted being “shocked that people were still coming out in groups of people to have brunches, to come out and enjoy food. I made the decision, because I felt myself getting a little obsessed with my customers. I couldn't believe that people were still going out and enjoying and doing, and I was encouraging it as a business owner.”

The protection of staff and customers and the mitigation of the public health crisis were cited by all businesses that closed early, and came up, in situation-specific forms, in almost all the other interviews. Businesspeople felt that they had a “responsibility” to the community (this came up in many interviews; e.g., US/Washington, food services; US/Georgia, food services; Canada/British Columbia, fitness services). Indeed, when pressed about why she chose to close early and to forego profit, the California business woman who felt staying open was “selfish” said that businesses “were more about community [than about profit].” For her, the motivation behind her business is not solely financial.

At the same time, decisions to close were not only based on moral and public health considerations. All but one of the businesses that closed early mentioned that business had decreased precipitously even before they decided to close. (And the one exception is a fitness services business in a part of Canada where there were comparatively few COVID-19 cases, whose owner decided to close early and shift to online classes after her husband went on a business trip to Italy). Indeed, the California business owner who felt that business is about “community” told the story of one of her “locals” coming in to her store on the last day she was open: “Even on the last day that I closed, one of my locals came in, they were walking and bought some stuff, but it was so slow that it was pointless to be open. I was stressing out because it was like, 'Okay, I'm so slow and I'm sitting here and I'm stressing out.' I didn't really know. I guess it's an unknown thing and it's more easily transmitted and all that, so I just decided [to close].” Her stress about the growing public health crisis and the threat it posed to herself and her staff, combined with decreasing financial payoffs for staying open, led her to close her brick-and-mortar shop and shift her business online.

We see the same from a Georgia food services business that similarly closed before being mandated to do so:

In 12 years, we’ve never closed for more than a few days at a time. It was hard because we didn't want to have to, in a sense, like—there was a big sense of the unknown like nobody knew where we were going to get help from whomever for our bills and our employees, and their own lives, their own bills and stuff. That part was very scary. There wasn't again very much guidance. But we had already started seeing like a drop-off in business anyway. People were paying attention to what was going on….If you couple that with the fact that we don't want to be part of the problem. If this will be a thing that shuts things down for a time, then we should be part of the group of people who decide to listen to the science and just shut down. Hopefully, in doing so we are able to curb the worst of the pandemic and reopen sooner than later.

Here, we see the business owner explain the choice to close both in terms of the “drop-off in business” and the community-focused desire to not “be part of the problem” and instead help curb the pandemic. Thus, rather than one or the other, both financial and public health considerations entered into focus when making the choice to close.

### Economy versus public health: “As one goes, so goes the other”

Generally, economic and public health justifications were intertwined, even when looking beyond the matter of closing. When asked for their “thoughts on the matter of public health versus the economy,” interviewees’ first response was typically to note that it is a tough question. The answers that followed hit upon both public health and economic considerations, in more or less overt ways.

In one of the starkest examples, one Washington businessperson, who was forced to permanently close his food services business, put it as follows:

So that's a tricky one to answer, right? If you answer it one way everybody looks at you like you don't care about human life, which we all care about…but at the same time…like I mentioned about our business, it’s a living, breathing entity [involving] food sales, right, and generating revenue and you can't just shut things off and expect [it] to be okay. So you're basically saying, you know, how many deaths are okay to allow our economy to continue to be strong and that's where, you know, I think the government did a good job of making sure that people stay safe. And then I think their thought was well, let's protect lives and then we'll figure out what to do with the economy later, which is okay. I mean, I think they've done a good job, but at the same time it sacrificed businesses, and it's going to continue to do so…all these people are unemployed. They're going to lose their health insurance. They're going to lose their ability to put food on their table, to pay their rent.

Thus, even someone suffering substantial, long-term financial consequences from COVID-19 did not elaborate a black-and-white choice between health and the economy. Instead, he acknowledged that the matter is a difficult one, and can be easily interpreted in starkly moral terms: if you privilege the economy, “you don’t care about human life.”

And indeed, this respondent well knows the effects of closing the economy. Elsewhere in the interview, he mentioned that while he had always wanted to be an entrepreneur, and this was not his first business, he was reconsidering his choices and wanted to have a less risky job in the future, for the sake of his family. The failure of his business—“a living, breathing entity”—was therefore deeply felt. But at the same time, he insisted that the government had done “a good job” in protecting people rather than focusing on the economy.

The end of the quote above further shows his view that the economy and public health are interrelated. While the government has “done a good job” in shutting down the economy, this had “sacrificed business” and led to considerable unemployment. The result of this economic downturn, however, was partially expressed in terms of public health: the unemployed will “lose health insurance.” The respondent, then, was clearly struggling with knowing the morally correct reaction to the pandemic, feeling torn and uncertain, but ultimately elaborating a polysemous view of the crisis happening around him.

Such views, as mentioned, were common throughout the interviews. The owner of a Georgia food services business designated public health versus economy as “a false choice,” noting that “there's no debate for me. It's like you cannot have one before you have the other.” As another Washington food services business owner succinctly put it, “there is no healthy economy without public health.” Or as argued more starkly by a different Georgia food services owner, customers are “not going to buy anything if they're all gone [i.e., dead] or in the hospital.”

Businesspeople also noted that this was not a COVID-specific debate. One co-owner of a Washington food services business expressed his long-held frustration with the “black-and-white” distinction made between health and economy, particularly among businesses:

Do you know, in the old days, meaning eight weeks ago [i.e., before COVID], people used to debate the same thing. I don't think they were as overt in discussing it this way, but people used to pit public health against the economy. It sounded like this. It said, well, in one company, the bottom line is the most important thing, and in another company, the people are the most important thing, and often those are in conflict. I would say that the bottom line and the people are not in conflict with one another and I would say that to our government, I would say the economy and the health of the nation are not pitted against one another, they are dependent upon one another. Just to say, as one goes, so goes the other….It's just so much more nuanced than that. I would just love to say to the world, “newsflash, your black-and-white world is a false dichotomy; it's just way more gray than you want it, than you care to admit, or you care to believe, and I know it's a lot of work to understand the gray.”

This businessperson therefore suggested that while the debate over public health versus economic well-being had become starker during COVID, businesses had long had to locate themselves on the spectrum between “the bottom line” or “the people.” But whether before or during COVID, the respondent felt that “the world” generally subscribed to a “black-and-white” view of economy versus public health.

Indeed, the polysemous view of the crisis was self-consciously described by one Georgia businessperson as “naïve,” even as he argued the black-and-white debate over economy versus public health was “counterproductive”:

This is going to probably sound a little bit naive, or like I'm a hippie or something, but I am not thrilled at the idea that those two things have to be mutually exclusive. I think that as creative as we have been in trying to make up the difference, I think that people should be more willing to be flexible....I think that putting those two things in opposition to one another causes people to have to choose one or the other. I think at a time where we should be taking care of each other and our neighbors, to be argumentative in that way is counterproductive.

But of course, reflecting widespread public debates on these matters, some of the interviewees were more starkly for or against privileging public health before the economy. However, even those who expressed skepticism about government mandated closures (about 15% of the sample) did so regarding the shape of public health measures rather than their existence. The respondents who expressed strong skepticism all felt that closing down the economy is not a feasible long-term strategy, and so governments should have taken long-term measures to protect those at risk while maintaining some level of economic activity.

For instance, the co-owner of a food services business in Virginia insisted that she “didn’t fall squarely in either camp [i.e., privileging public health or the economy]” but also argued that “at some point, we are going to have to accept that we can't close the global economy for a year and a half until there's a vaccine. We will have to accept some level of death, I think, from this.” In particular, she noted that “there will probably, at some point in 2020, need to be a shift to saying to folks who do have underlying health risk, like, ‘You are the folks that need to stay home, and the people who might be asymptomatic or a little bit healthier are the ones who are going to have more freedom.’”

On the other end of the spectrum, even those in favor of a strict and prolonged economic shutdown for the sake of public health eventually came back to economic matters in their answers. For instance, the CEO of a California manufacturing company, who likened those in favor of reopening the economy against public health guidelines to “[people] who really [believe] the Earth is flat” insisted that those protesting for reopening should instead be expending their energies in more financially efficient ways:

I understand you want to take the shortcut and just all the effort that you're putting into protesting and making these ridiculous signs and shouting and then commenting on every social media platform. You know, it's a lot of energy and work. You could spend all that time going through the government websites, figuring out how to get money for your company....Forcing the most obvious thing, which can’t be done, reopening the economy, this just seems like a waste of energy, you know, and you're going to put in all this creativity of writing these signs, and coming up with clever comments on Facebook. Like, I don't know, save that creativity and energy to write your next value proposition for your pivot.

To this respondent, then, the most important problem posed by the protesters was their financially inefficient use of time. Because, according to this respondent, reopening the economy “can’t be done,” time spent protesting would be better spent working on responding and adapting to the crisis.

### Considerations in reopening: “I can’t afford it, but I have to be afraid for my life on top of that”

The nuance in businesspeople’s views of the interplay between financial and health matters is especially evident among businesses who had the option to reopen, but chose not to do so. Indeed, this phenomenon was widespread, as seen in statistics on spring and early summer economic activity in states such as Georgia (Schneider 2020). Like closing voluntarily, choosing not to reopen was also the subject of highly fraught decision-making.

Among the businesses that chose to remain closed, we again see a view of business as about more than profit. For instance, one Georgia food services owner noted that he did not start his business for the financial payoffs, having left a job with higher income. As he put it, “my motivation was not income. My motivation was community, basically.” This included his employees, customers, and “the larger community overall.” It is from this perspective that the COVID-19 pandemic had been difficult for this respondent: “it’s been very testing over these times, more for the safety factor. What is safe to do, what's right for the community, for my staff and the community to do, rather than what's right for my business.” This meant deciding not to reopen his business: “it's because people want that comfort, they're willing to ignore the science and live with some illusion of comfort, so I don't see the [business] opening.”

But this did not mean that financial considerations were irrelevant to this business owner. His aim was paying attention to “what it safe,” which was part of the decision not to reopen. But he also elaborated an extensive financial plan for staying afloat during the crisis. For him, beyond safety, the crisis involved “at the same time looking at what is it going to take to be [financially] successful in this model, you know. In other words, not giving up.”

Indeed, while decisions to not reopen pertained to public health, they also had important economic bases. One Georgia business owner mentioned that the morning in late April when we spoke, he had driven around his city and there was “nobody on the street. There's no traffic out there. It's still pretty damn quiet.” This was after the governor of Georgia allowed businesses to reopen. But for this business owner, no people on the street meant no customers were he to reopen his business. His plan was therefore to watch other businesses who had reopened to see if and when customers came back, and to only reopen then.

In fact, this was a common view, that reopening would mean losing money, given higher operating costs but no customers. As put by a different Georgia food services business owner: “if we’re going to do it, we want to bet the odds [so] that we have a better chance of being successful. [These better odds will] be later dates than we had rushing out here just to do something now to lose money for the next two or three weeks when you know, when people are still getting sick, when people are still not protecting themselves.” And, as he had mentioned earlier, with schools not open and unemployment growing, that meant less disposable income for customers to spend in his business. He did not believe that customers would be coming back, when their mentality would be that “I can’t afford it, but I have to be afraid for my life on top of that.” His goal was therefore to wait until consumer confidence had grown in order to reopen.

Business owners who chose not to reopen generally had little trust that customers would return right away, particularly because they felt economic reopening had happened too quickly, and the dangers involved in going to restaurants or shops were still considered too high. Indeed, one Georgia food services owner argued that it would have been better to have a more drastic shutdown “like in Spain, even for a week or two.” The lower infection rates resulting from such a shutdown, he felt, would have meant higher consumer confidence upon reopening.

Beyond the expectation that customers would not return, the other factor involved in economic considerations to reopen was directly related to the feedback loop between public health and economic well-being, namely the possibility of a spike in infections that would lead to another closing. Indeed, even in contexts where reopening was not yet allowed, respondents spontaneously mentioned that needing to close again would be devastating, both in terms of morale, but also financially. As a Georgia businessperson starkly put it, “if you close again, you never reopen.” (For instance, a Washington food services business estimated that it would cost them about $5000 just to stock up with necessary supplies for reopening, supplies that, because they are perishable, had been thrown out when closing was first mandated. They believed they could with difficulty afford to restock once, but any more than that would lead them to bankruptcy.) Indeed, as a result of the costs of reopening, one Georgia food services business mentioned that they had decided to remain closed for two weeks after the official reopening date, precisely to wait out the COVID-19 incubation period. Their plan was to reopen after these 14 days only if infection rates had not spiked in Georgia.

There were two other economic considerations mentioned by business owners. The first was the possibility of lawsuits resulting from improper precautions leading to COVID-19 infections in their business: lawyers, according to one business owner, were already “lining up” (Georgia/USA, food services). Along similar lines, a different owner brought up the “negative publicity” of such an event, which he imagined could “sink” his business permanently (Georgia/USA, food services).

Finally, there is some evidence from the interviews that such economic motives for remaining closed also served an important function in underscoring that decisions to not reopen are “not political” (Georgia/USA, food services). Two business owners mentioned that to them, the fact that reopening made little financial sense was enough to escape the heated political debates on reopening. Simply put, “they’re telling me to be open and to make sure I'm paying my sales tax and doing all the things I need to do…but then they've limited the number of people that can come in to the [business]” (Georgia/USA, food services). In businesses with small margins such as food services, this, it was felt, was simply not financially feasible.

So, as another Georgia food services business owner put it, the decision not to reopen “wasn't political…it was just like hey, this is too early. You’re making the wrong decision.…We work on small margins, but we work to cater to lots of people and that's how we do our business. But…we are the backbone of our communities…we keep communities together….” This is why losing many small businesses would be “devastating,” and why rather than hasty reopening, he argued that more expansive loan programs were urgently needed. “We’re not in this to make money. That’s a byproduct of it, yes. But you’re in the business to take care of people, and you have to love it. You know, you have to love it.”

## Conclusion

The widespread economic effects of the coronavirus pandemic posed substantial challenges for businesses. Indeed, with governments choosing to lift restrictions, sometimes against the advice of public health experts (Lash and Wezerek 2020), the individual choices of businesses on when and how to engage in economic activity could have important effects on public health. Interviews, meanwhile, painted a picture more complicated than models of self-interested profit maximizing would suggest: business decisions on how to react to the crisis typically had financial dimensions, but profit was not the sole motivation. For people in business, economy and public health were intertwined as part of polysemous views of the crisis and of their responses to COVID-19. Rather than privileging one over the other, a wide range of businesspeople—including many struggling to survive the crisis—saw them as interconnected.

In the absence of a survey mechanism, it is not possible to determine the extent to which the interviewees are a representative sample. In particular, it is possible that the businesspeople most struggling with the effects of the pandemic were less likely to participate in the study. More research is certainly needed to better determine the range of experiences during COVID. It should be noted, however, that many of the interviewees confessed to struggling greatly during the crisis, and indeed many continued to work as the interview was taking place. At the same time, interviews also included businesses thriving as a result of the pandemic (because they offered goods that were suddenly in high demand as a result of COVID-19), and it was across this wide range of businesses that polysemous justifications were employed to make sense of the pandemic.

A drawback of this work, however, is the lack of precision in delineating the fields within which these people in business were justifying and (re)orienting their behaviors (Rawlings and Childress 2019; Spillman and Strand 2013). Luft, for instance, argues that “in challenging and uncertain times” (2020: 1), situations and relationships shape moral cognition. And there is certainly some evidence of this in the interviews. The California businesswoman mentioned above closed early because the COVID-19 situation was causing her anxiety, because she felt staying open was “selfish” *and* because she “saw some of the other businesses just shut their doors.” On the other hand, national context (at least when limited to the Canada-US comparison; see the Appendix) seemed to have little effect on how businesspeople were making sense of the crisis. Future research could certainly delineate the social contexts in which actors navigate the COVID pandemic, and even specify which contextual factors hold sway for people in business (though see Demertzis and Eyerman 2020, Lo and Hsieh 2020, or Morgan 2020 for such considerations).

### Directions in conceptualizing crisis cognition

Interviews with 60 people in business suggest that the COVID-19 crisis did not consistently limit cognition. This could indicate that psychological models of circumscribed cognition only focus on a narrow band of the larger crisis moment. And this has, indeed, been a subject of active debate among psychologists. As Meder et al. (2013: 259) argue, “real-world problems like economic crises highlight the potential limitations in the way decision-making behavior is usually conceptualized in both economics and the cognitive sciences, particularly with respect to the many forms of uncertainty that people face outside the laboratory.” In particular, concerns exist that studies of decision-making might inadvertently translate uncertainty into risk, effectively assuming that the unmanageable is, in fact, manageable (Osman et al. 2012).

The discrepancy between generative and reductive views of crisis could therefore be explained in terms of sequencing or scope, such that psychological studies of decisionmaking under conditions of uncertainty have a narrower focus, towards a singular actor in the first moments of crisis. In contrast, generative conceptions of crisis could be seen to focus later in the crisis moment, and beyond the scope of the single individual juggling risky options in a laboratory setting. In this sense, the modified generative conception of crisis elaborated here—the polysemous justifications employed by businesspeople making sense of COVID-19—is simply a real-world example of behavior during a *later* stage of crisis, when businesspeople have had the chance to think through the pandemic.

But this is, at best, a partial explanation, even in psychology. Studies in the naturalistic decision-making tradition are predominantly focused on real-world scenarios of decision-making, showing that even as experts are generally well able to manage common types of uncertainty, they are ineffective at navigating moments of crisis, of shifting sociocultural scaffoldings for action (Kahnemann and Klein 2009). Meanwhile, research on climate change shows that even after years of knowledge on how best to address a crisis, uncertainty continues to limit cognition on the issue (Gifford 2011; Gifford and Chen 2017; Lacroix and Gifford 2018).

Thus, an alternative explanation for the discrepancy between generative and reductive views of crisis cognition might rest in imprecise—and inequivalent—conceptions of cognitive complexity. Theorizing on the transformative and generative nature of crises (Bourdieu 1990, 2000; Butler 1993, 1999; Foucault 1997, 2000; Swidler 1986) insists on a greater explicitness of culture during unsettled times. But there is little sense of what these heightened levels of articulation might look like.

It is here that this paper intervenes, elaborating justifications as an easily operationalized mechanism through which crises become thinkable. Multiple justifications can therefore be suggestive of heightened levels of both cultural articulation and cognitive complexity, as actors seek to make sense of the crisis moment. And indeed, the interviews show that even against a background of polarization on the subject—after all, the stark choice between public health and economic well-being drove the 2020 US election (Medina and Russonello 2020)—businesspeople elaborated complex views of the interplay between the two.

But future work is needed to better elucidate the conceptual and methodological relationships between the generative and reductive views of crisis. The conceptual model elaborated here is only a first step in this direction. The interviews show that businesspeople justified their actions in multifaceted ways that expand well beyond profit motivations, just as they do in more settled times (Boltanski and Thévenot 2006 [1991]). These empirical findings therefore gesture to the power of diverse justifications in shaping actors’ motivations and actions (Boltanski and Thévenot 2006 [1991]; see also Levi and Sendroiu 2019), and in particular foreground how a plurality of justifications can underpin action in response to crisis moments. But future work should further explore how, and the extent to which justifications capture cognitive complexity.

### Morality, agency, and politics during crisis

In her account of sense-making among people in business, Spillman (2012) details how moral, normative considerations give meaning to economic, profit-seeking behaviors. Interestingly, here we in fact see the reverse, with economic sense-making helping to give meaning to (or more precisely to substantiate and justify) moral, prosocial behavior such as closing early or refusing to reopen. In a way, this in fact speaks to the strength and prevalence of profit-first narratives, though of course to ends considerably more complicated than profit maximization, foregrounding “interest articulation as a (sometimes destructive) rationalization of the search for solidarity” (Spillman 2012: 182).

In particular, there is some evidence in the interviews that political polarization was shaping the justificatory work done by people in business, even in the early moments of the crisis, and notably that using economic justifications was an attempt to escape charges of political polarization. This suggests that justificatory work can be political in ways that were perhaps not envisioned by Boltanski and Thévenot when they rejected Bourdieu’s focus on how power structures shape how individuals make sense of the world around them (see also Strand 2016; Stark 2011). The interviews presented here mostly bracket off the initial politics of crisis—the politicized debates on whether COVID-19 constitutes a crisis—instead focusing downstream after an individual has decided they are in crisis, with respondents invited to reflect on decision-making *during* crisis. It is therefore left to other research to determine and problematize the accession to COVID-19 as crisis. But even beyond the politics of acceding to crisis, the research presented here shows that thinking through a crisis moment involves polysemous justifications that may be underestimated if we only focus on models that detail how crises limit thinking. Indeed, the political polarization regarding economic activity during COVID-19—polarization that drove electoral behavior during the 2020 election (Medina and Russonello 2020)—may have obscured the extent to which businesses would not open even if allowed to do so, due to both public health concerns and perceptions of limited financial payoffs of being open.

In the case of COVID-19, businesses perceived that they would be affected by the pandemic no matter whether the economy was officially open or shut. Indeed, the interviews suggest that the most effective encouragement for economic activity would have been lowering the perceived risks of going to shops, restaurants, and so on. Because economy and public health are intertwined—and perceived as such by businesses—bringing the public health crisis under control would have likely worked best to improve economic activity.

The findings presented here thus have important implications for models of crisis that often define emergency moments through an abrogation of agency, deliberation, or thinking. Such models, according to Elaine Scarry (2011: 81), are based on “the belief that action requires putting aside thinking,” especially the *rapid* action that an emergency situation mandates. The result, in turn, is that crisis can be a concept to “conjure with,” a potentially transformative cultural construct (Roitman 2014). But the meaning of a crisis is in fact far from obvious (Alexander and Smith 2020; Reed 2015; 2016), the subject of performative contestation through a multiplicity of justifications which render the crisis moment thinkable.

## Note on the split Canada/US sample

The businesses interviewed operate across a range of policy contexts not just nationally, but regionally. Indeed, a respondent from Texas decried how divergent enforcement protocols at the county level meant that a business similar to his could stay open just a 15-minute drive away. On the other hand, when considering national differences, the two policy contexts seemed to have little effect on businesses’ early experience of the crisis. While Canadians were less likely to complain about how their federal and provincial governments had addressed the crisis (and some were even openly complimentary), businesspeople in both countries were stressed and confused about government financial help, and particularly worried about government rules being confusing, unclear, and unpredictable. Moreover, while Canadian governments moved faster to restrict economic activity, the interviews involved businesses that closed early in both countries.

The one divergence was around refusals to reopen. Two Canadian businesses, in Nova Scotia, were in the midst of reopening when interviews took place, whereas interviews were done with seven Georgia businesspeople refusing to reopen despite being allowed to do so. This divergence could be explained in terms of sampling and interview timing. In late spring when the interviews took place, the Canadian reopening was more gradual and delayed compared to American states such as Georgia. Moreover, both Nova Scotia businesses that were interviewed were offering leisure services that are seasonal in nature, meaning that their experience of the crisis was different from the predominantly food services businesses interviewed in Georgia. It should nonetheless be noted that both reopening Canadian businesses were deeply skeptical about the future, and about their longer-term abilities to pull through the crisis.

Altogether, there is certainly a need for more data, especially data that are nationally representative and longitudinal, in order to better detect the effects of divergent policy environments on how businesses coped with the COVID-19 crisis. The goal of this project, however, was to excavate the ways in which businesspeople motivate, justify, and navigate their responses to the crisis. These justificatory strategies, the interviews show, are remarkably similar across the two countries, speaking to the polysemous justifications employed by both American and Canadian businesses during COVID-19.

## Footnotes

1 These multifaceted business identities fit within the broader context of research on prosocial behaviors, which typically focuses on the individual factors that produce prosocial behavior in contexts where self-interested behaviors would lead to collectively suboptimal outcomes (Simpson and Willer 2015). The large literature on such social dilemmas from psychology (Batson 2011; Eisenberg et al. 2002), behavioral economics (Fehr and Gintis 2007), and evolutionary biology (Gintis et al. 2003; Sober and Wilson 1999) shows that individuals have prosocial motivations and take prosocial action.

2 Research shows a similar sort of cognitive narrowing in criminal justice sentencing. In order to reduce complexity and more effectively clear large caseloads, judges narrow in on a few issues when making sentencing decisions (Steffensmeier et al. 1998; see also Johnson et al. 2015; Leiber and Peck 2015; Logan et al.^2017^; Stemen et al. 2015). In turn, this sort of focal concerns sentencing has been linked to disparities in sentencing outcomes: “[b]ecause judges typically operate under time constraints and under conditions of uncertainty, often with incomplete information about defendants, they may make stereotyped associations between defendants’ demographic characteristics and the three substantive concerns in their decision-making” (Lynch 2019: 1149). This framework is therefore tightly linked to social psychology research on heuristics and stereotypes (Lynch 2019; Steffensmeier et al. 1998).

3 It should be noted that in subsequent work, Boltanski (2012) argued that certain areas of life defy these polysemous justificatory orders and are in fact governed by power relations. However, inasmuch as *On Justification* arose out of an attempt to explain “controversy” (Friedland and Arjaliès 2017), it is this work I primarily draw on here.

4 Indeed, their view of crisis is quite radical, arguing that crisis has little to do with action, rather seeing crisis as a *precursor* to action, defined instead through the meaning of reality being up for debate. As they put it, “[t]he crisis is therefore a paradoxical moment in which, unlike the moment of action, the question of agreement about reality occupies everyone’s mind but in which, in the absence of realization in a present filled with commitments and expectations, the sense of reality is lacking” (Boltanski and Thévenot 2006 [1991]: 350).

5 See, for instance, Carruthers (2013) for the distinction between uncertainty versus risk. In essence, risk is measurable uncertainty, a conception that is often attributed to Knight (2014). As a result, uncertainty—sometimes called Knightian or ontological uncertainty—is better related to crisis (defined as moments of widespread uncertainty when shifts in cultural scaffoldings render the effects of behavior unpredictable (Bourdieu 1990; Lizardo and Strand 2010; Swidler 1986)). In this sense, turning uncertainty into risk is effectively an attempt to manage the crisis, and move beyond it.
